# Cylindroma of the Breast: Case Report of a Rare Breast Neoplasm

**DOI:** 10.7759/cureus.69896

**Published:** 2024-09-22

**Authors:** Michail Angelos Papaoikonomou, Aggeliki Chlorou, Europi Michailidou, Gregorios Panselinas, Maria-Eleni Michailidi

**Affiliations:** 1 Department of Surgery, Agios Pavlos General Hospital, Thessaloniki, GRC; 2 Department of General Surgery, Agios Pavlos General Hospital, Thessaloniki, GRC; 3 Department of Pathology, Agios Pavlos General Hospital, Thessaloniki, GRC

**Keywords:** adenoid cystic carcinoma, breast lump, breast screening, breast tumor, cylindroma

## Abstract

The majority of breast malignancies are either ductal or lobular tumors. A rare benign neoplasm of adnexal origin, cylindroma of the breast, was first described 23 years ago, and 21 cases have been documented in the literature since then. We report a case of a breast cylindroma on a 62-year-old woman who presented with a nodule first detected by a national mammographic screening program. We review the radiological and histological characteristics, diagnosis, and clinical course of this entity and discuss the previous cases in the literature. In addition, we emphasize on the challenges of differential diagnosis of this rare form of breast tumor from the solid variant of adenoid cystic carcinomas and the management of similar conditions.

## Introduction

Cylindromas are benign tumors of adnexal origin, which commonly occur on the head and neck as a solitary nodule or multiple lesions. Cylindromas have also been reported at other extracutaneous sites including the salivary gland, bronchus, lung, breast, and kidney [1-2]. Breast cylindroma is a rare benign neoplasm that resembles benign dermal cylindroma in both morphology and immunophenotype [3-4]. Since its first description in 2001 [5], a total of 21 cases of breast cylindroma have been reported in the literature so far. We report a case of a 62-year-old woman with a cylindroma of the left breast. In addition, we highlight the importance of breast screening for early detection of this condition and the challenge of differentiating breast cylindroma from the solid variant of adenoid cystic carcinoma as the management of these tumors is different.

## Case presentation

**Figure 1:**
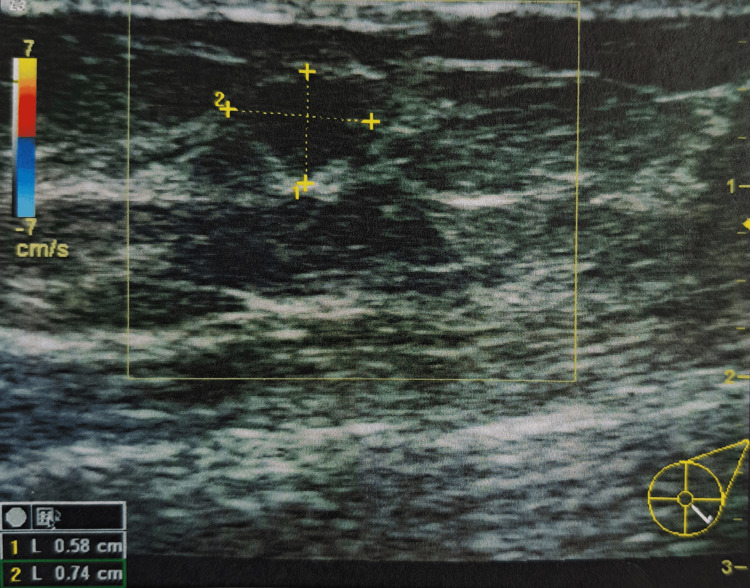
Breast ultrasound showing a hypoechoic nodule 0.6 x 0.53 x 0.74 cm with lobulated borders, at the four o'clock position of the left breast

**Figure 2:**
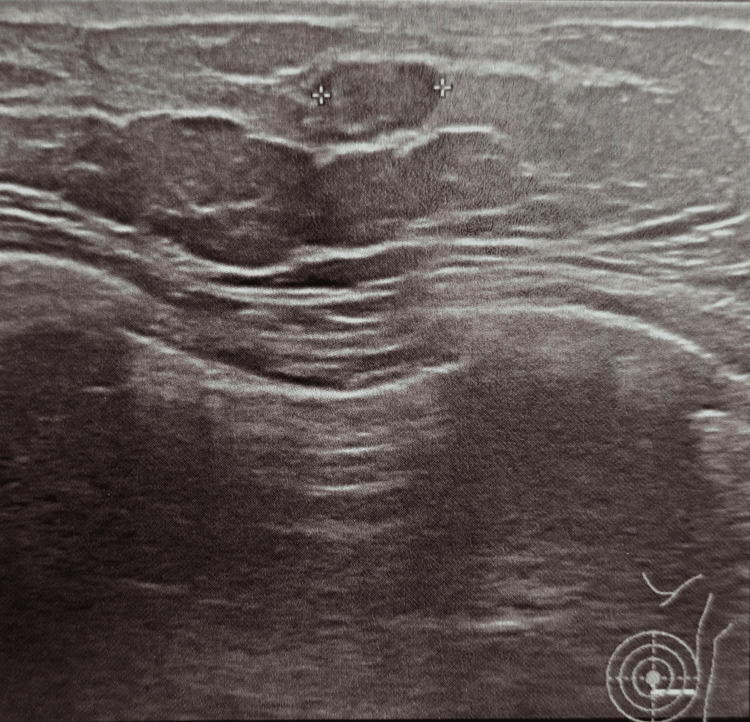
Breast ultrasound showing the hypoechoic nodule which has increased in size from 7.5 mm to 11.6 mm on the left breast

**Figure 3:**
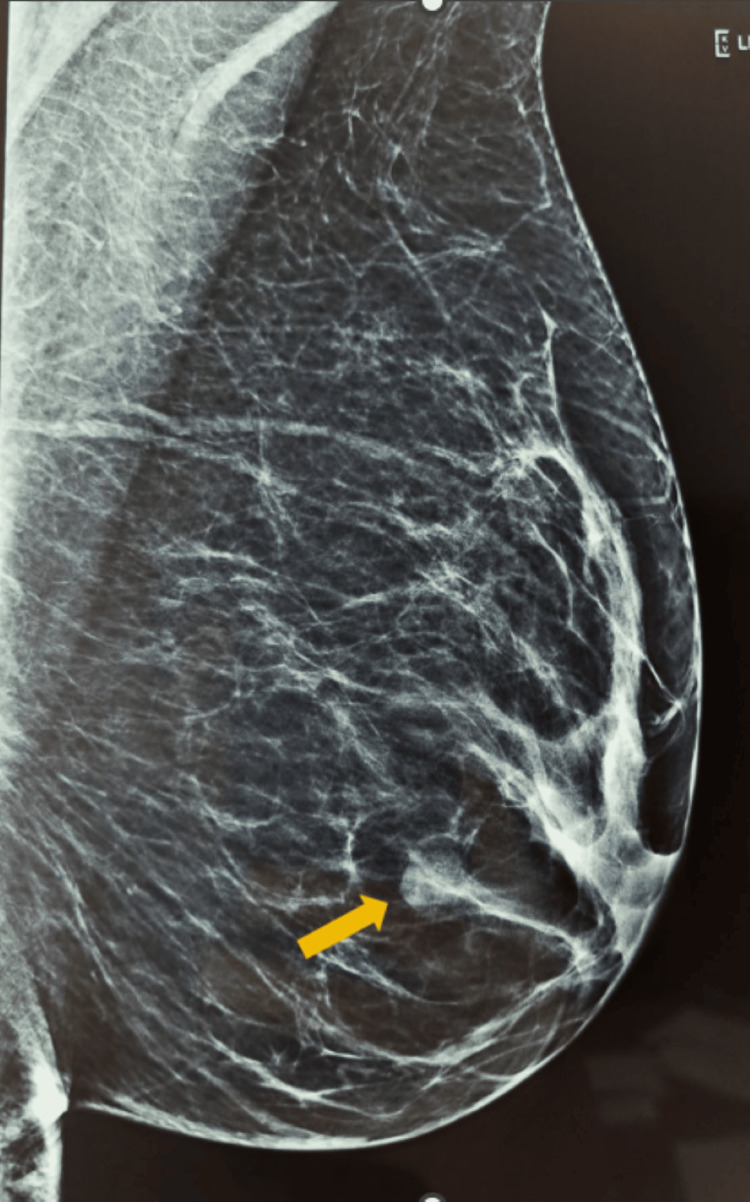
Mammography of the left breast showing a well-defined nodule in the lower outer quadrant of the left breast

A 62-year-old female patient visited the Outpatient Breast Department of our hospital due to a growing nodule of the left breast. The nodule was not palpable; there were neither skin nor nipple changes. Axillary lymphadenopathy was not detected. The patient did not refer any significant medical disease and has no family or personal history of any breast lump or breast cancer. The nodule was first detected three years ago during screening. The mammography showed a 7.5 mm nodule with well-defined borders of the lower outer quadrant of the left breast, next to the areola. The ultrasound imaging confirmed the presence of a hypoechoic lesion at the four o’clock position of the left breast. The lesion was characterized as breast imaging reporting and data system score (BIRADS) 2, and the patient was advised to continue the annual screening. During the follow-up, the nodule increased in size from 7.5 x 5 mm (Figure 1) to 11.6 x 6 mm (Figures 2, 3).

**Figure 4:**
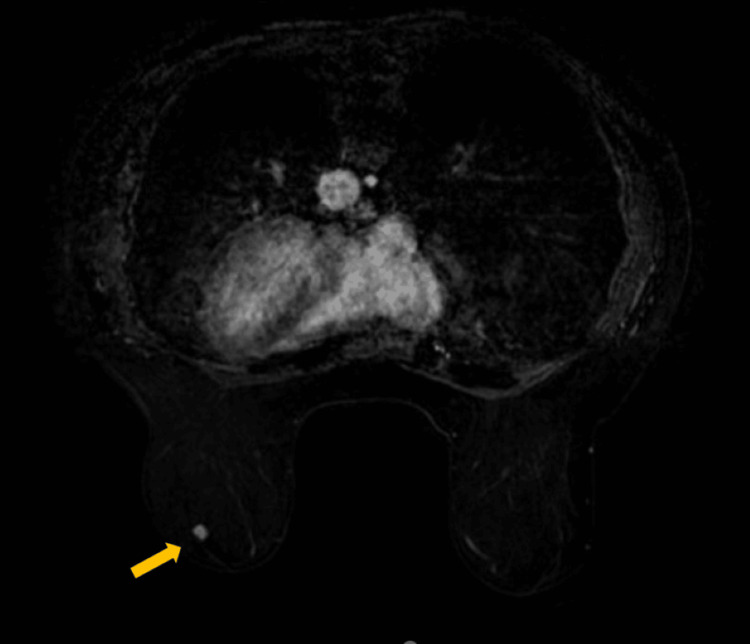
Contrast enhanced T1-weighted fat suppressed MRI image of the left breast showing a nodule with lobulated borders and type II pattern of enhancement. The arrow indicates the homogeneously enhancing oval mass on the lower outer quadrant of the left breast MRI: Magnetic resonance imaging

In addition, the lesion was characterized with heterogenous echogenicity and internal vascularity. The lesion was subsequently classified as BIRADS 3. Further investigation was considered necessary, and magnetic resonance imaging (MRI) was performed. MRI showed the nodule with lobulated borders and type II pattern of enhancement (Figure 4). Thus, the lesion was classified as BIRADS 4.

**Figure 5:**
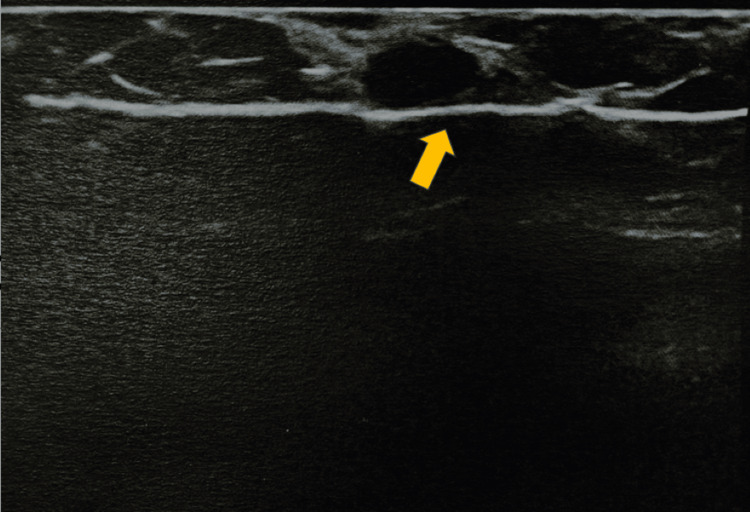
Intraoperative breast ultrasound of the surgical specimen after hookwire-guided wide local excision confirming the excision of the suspected lesion

Core biopsy was suggested, but the patient denied it. Therefore, an excisional biopsy was performed. The patient underwent surgery with wide local excision after an ultrasound-guided hookwire placement. The surgical specimen was sent for ultrasound imaging intraoperatively (Figure 5) in which the complete excision of the lesion was confirmed, and then the specimen was sent for histological examination. 

**Figure 6:**
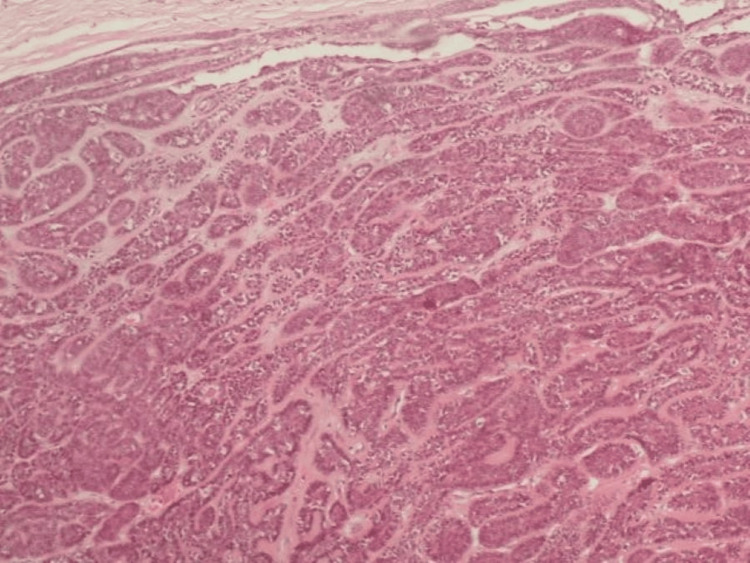
Biopsy specimen. H & E stain. Nests of basaloid cells surrounded by hyaline membrane, showing the typical ”jigsaw” pattern, containing a dual population of larger, eosinophilic, epithelial cells in the center and smaller, basaloid, myoepithelial cells in the periphery H & E stain: Hematoxylin and eosin stain

**Figure 7:**
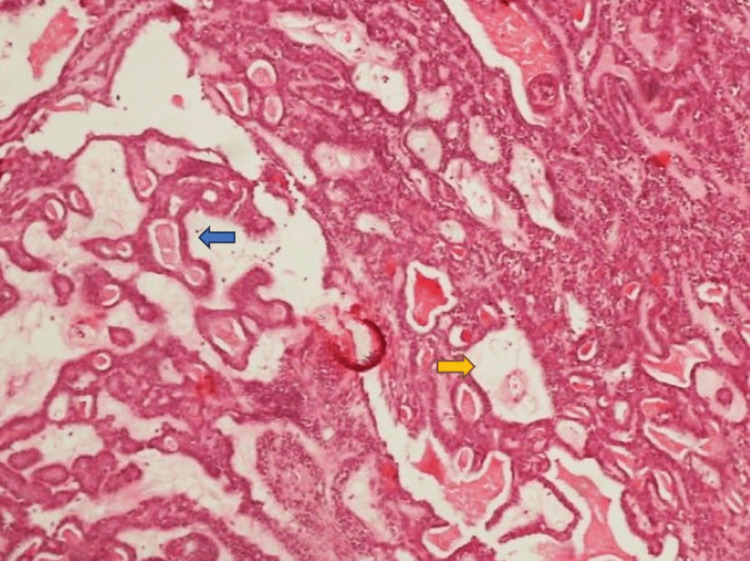
Biopsy specimen intermediate power magnification. H & E stain. Nests of basaloid cells lacking significant atypia surrounded by a thickened basement membrane (blue arrow) composed of hyaline droplets (yellow arrow) H & E stain: Hematoxylin and eosin stain

Histological sections of the surgical specimen were stained by hematoxylin and eosin. On microscopic examination, the cylindroma showed a total size of 0.9 cm with irregular border. The tumor was located within the breast tissue and was not attached to the overlying skin. It consisted of inner epithelial and outer myoepithelial cells in tightly packed cell islands forming the typical mosaic/jigsaw pattern. Occasional ducts, indistinguishable to those of normal breast tissue were also noted within the tumor. Immunohistochemistry staining was performed, and the central basaloid cells were CD7-positive, while the peripheral myoepithelial cells were p63-positive. Immunohistochemistry was negative for estrogen receptor (ER), progesterone receptor (PR), and human epidermal growth factor receptor 2 (Her2). Mitoses were rare, while necrosis, atypia, or nuclei pleomorphism were not present (Figures 6, 7).The patient’s postoperative course was uneventful, and routine follow-up was arranged. The tumor had not recurred six months postdiagnosis.

## Discussion

To summarize, we describe a case of breast cylindroma on a 62-year-old patient with a small, well-circumscribed nodule on her left breast first diagnosed during the annual mammography three years ago. During the follow-up, it increased in size requiring further imaging with MRI in which the lesion was considered to be suspicious, given the BIRADS 4 score. As in other cases, a core needle biopsy would have been performed in order to evaluate this lesion in greater detail but the patient declined. Excisional biopsy after hookwire placement was decided to obtain the histogical diagnosis of the lesion, and the patient's postoperative course was smooth. On microscopical examination, the tumor exhibited histological and immunohistochemical characteristics of a rare breast cylindroma, namely, consisted of tightly packed cell islands of inner epithelial CD7+ and outer myoepithelial cells p63+ forming the typical “jigsaw”/“mosaic” pattern. So far (Feb 2024), there have been no reports of distant metastases or recurrences in the literature, supporting the idea that breast cylindroma is a benign lesion. Therefore, it appears that axillary lymph node excision is not necessary [6]. In our case, we suggested a six-month follow-up in which the patient had no clinical or imaging findings of recurrence of the lesion. 

Cylindromas are relatively benign skin adnexal tumors occurring most commonly on the head and neck as a solitary nodule. Multiple cylindromas can be presented growing together in a hat-like configuration, called “turban” tumor [7]. Familial cylindromatosis or Brooke-Spiegler syndrome is a rare familial condition with autosomal dominant inheritance and the tendency to form adnexal tumors such as trichoepithelioma, cylindroma, and rarely spiradenoma. The lesions are present not only on the head and neck but also on the extremities and the trunk. This condition is associated with CYLD1 gene mutations [8]. The breast is considered a modified sweat gland which explains the occurrence of benign and malignant neoplasms resembling those originating from the sweat glands in the breast, such as cylindromas. Breast cylindromas are usually presented as a single, movable, well-defined mass within the breast. The tumor can be a few millimeters to several centimeters in size, and it has an oval or round shape. Although cylindromas are frequently painless, some patients may experience discomfort, especially when they are associated with spiradenoma [1]. While some cylindromas are observed close to the nipple's main lactiferous ducts, they are not attached to the skin that covers them. The skin that covers them could be normal or slightly thicker [1]. All of the reported cases were females, mostly older than 60 years old, and their age ranged from 37 to 80 years old. Cylindromas can be presented either during the breast screening or as a palpable mass of the breast. During the physical examination of these patients, there are no particular clinical indicators that would have led to the diagnosis of breast cylindroma. According to the current literature, they can also be found as incidental findings in surgical specimens from women with invasive breast carcinomas (two cases of invasive ductal carcinoma, one case of invasive lobular carcinoma, one case of an invasive grade 2 breast carcinoma of no special type) [9-10]. Imaging studies such as mammography, ultrasound, or MRI cannot differentiate cylindroma from other benign breast tumors; instead, they give the impression of a benign lesion without any other signs of malignancy. Mammography may reveal a well-defined mass with smooth margins and homogeneous density. Ultrasound may demonstrate a solid hypoechoic mass. MRI may reveal a well-defined mass with lobulated borders and type II enhancement curve. The diagnosis of breast cylindroma can be reached only by histopathological exam. Core biopsy [6] or fine needle aspiration [10] may be performed to help confirm the diagnosis histologically. The main histological differential diagnosis for breast cylindroma is the solid-basaloid variant of adenoid cystic carcinoma of the breast (AdCC) [11]. Both mammary tumors have been found to have eccrine ductal structures and hyaline globules of basement membrane [1]. Differentiating these two lesions can be challenging since they both consist of a dual population of CK7-positive central epithelial cells and p63-positive peripheral myoepithelial cells, and they are both triple negative tumors [9,11,12]. Important differences in their morphology include the presence of mitotic figures and atypia which are detected in most of the cases of adenoid cystic carcinoma of the breast [13]. The adenoid cystic carcinoma of the breast varies between two forms, the cribriform and the solid variant [14-15]. The cribriform variant is characterized by the following features: cribriform structures, cytological atypia, brisk mitotic activity, invasive growth pattern, and mucin secretion allowing it to be easily distinguished from breast cylindroma. Conversely, the solid form of adenoid cystic carcinoma may be mistaken for cylindroma, particularly in excisional biopsy specimens, since both tumors have similar nodular and trabecular patterns, basaloid cells, myoepithelial cells, and infrequently, squamous cells [16]. Another histologic feature of adenoid cystic carcinomas is the lack of Langerhans cells, which are a consistent characteristic of cylindromas [17]. Because of the implications for the prognosis and management of these lesions, a definitive diagnosis is required for breast cylindromas. The solid form of AdCC exhibits a more aggressive behavior and has the potential for both lymphatic and distant metastases and local recurrences which necessitates a more aggressive treatment [9,17-19].The treatment of breast cylindroma is typically surgical excision with clear margins [6-20]. Out of the 21 cases reported in the literature, 16 underwent local excision [6,9]. Close follow-up with clinical examination and imaging studies is recommended to monitor for any changes in the tumor size or characteristics.

## Conclusions

Breast cylindroma is a rare breast lesion that requires careful evaluation and management. The diagnosis of mammary cylindroma in a preoperative core biopsy should prompt excision with a margin. Only histopathological examination can make the diagnosis, and immunohistochemical staining is used to distinguish it from the histopathologically similar solid-basaloid variant of adenoid cystic carcinoma of the breast, as there are clear differences in the prognosis and treatment of these lesions. These patients should be closely monitored because of the lack of long-term outcome studies considering the borderline histologic features of cylindromas. Breast cylindroma remains a challenging clinical entity for physicians due to the necessity to fully characterize such lesions histologically in order to exclude the malignant solid variant of adenoid cystic breast carcinoma.
